# Robot Care Ethics Between Autonomy and Vulnerability: Coupling Principles and Practices in Autonomous Systems for Care

**DOI:** 10.3389/frobt.2021.654298

**Published:** 2021-06-16

**Authors:** Alberto Pirni, Maurizio Balistreri, Marianna Capasso, Steven Umbrello, Federica Merenda

**Affiliations:** ^1^ Sant’Anna School of Advanced Studies, Pisa, Italy; ^2^ Department of Philosophy and Educational Science, University of Turin, Turin, Italy

**Keywords:** applied ethics, care ethics, bioethics, robotics, care robots, autonomy, vulnerability

## Abstract

Technological developments involving robotics and artificial intelligence devices are being employed evermore in elderly care and the healthcare sector more generally, raising ethical issues and practical questions warranting closer considerations of what we mean by “care” and, subsequently, how to design such software coherently with the chosen definition. This paper starts by critically examining the existing approaches to the ethical design of care robots provided by Aimee van Wynsberghe, who relies on the work on the ethics of care by Joan Tronto. In doing so, it suggests an alternative to their non-principled approach, an alternative suited to tackling some of the issues raised by Tronto and van Wynsberghe, while allowing for the inclusion of two orientative principles. Our proposal centres on the principles of autonomy and vulnerability, whose joint adoption we deem able to constitute an original revision of a bottom-up approach in care ethics. Conclusively, the ethical framework introduced here integrates more traditional approaches in care ethics in view of enhancing the debate regarding the ethical design of care robots under a new lens.

## Introduction

Developments in robotics and automation technologies are rapidly changing many aspects of our lives. The field of (health) care has been no exception, promising many boons while also bringing about controversial ethical questions. This paper takes care robots for the elderly as an object of analysis, evaluating the existing literature on their ethical and responsible design. In particular, we aim to discuss the existent approach to the ethical design of care robots by Aimee van Wynsberghe (2012); van Wynsberghe (2013a); van Wynsberghe (2016) that relies principally on the work on care ethics by Joan Tronto while also exploring the viability of a care ethics approach that is fundamentally non-principled, such as those expounded by Tronto (1993), Tronto (2010) in view of possibly envisaging a conciliation between the two alternative proposals (§ 1).

Tronto argues that general principles are too broad to constitute a sufficiently stable justification for actions consequent to care ethics. However, in recent years, the literature on care ethics has been trying to identify principles that can have an informative and a justificatory role in making moral decisions and carrying out moral actions in care practices (Collins, 2015) (§ 2). Though such an approach constitutes just one of the many different understandings of care ethics, it becomes particularly relevant as a theoretical basis for care robot programming, that is when the ultimate goal of philosophical research is to program machines able to interact with human beings in ways that are acceptable from a care ethics perspective. To this end, this paper explores the possibility of integrating 1) a care-ethical perspective based on the consideration of particular care relationships, their contextual levels and the importance of needs, emotions and sympathetic modes of deliberation with 2) a principlist approach to care.

Such an approach asserts that orientative principles, rather than constitutive ones, may have a justificatory role in grounding proper forms of action and would constitute one to be included in the category of the so-called “hybrid approaches” similar to the one proposed by Van Rysewyk and Pontier (2015) but with substantial differences that will be explored. According to a Kantian approach, the proposed principles are argued to be not mutually exclusive and contribute to identifying a more comprehensive account of care ethics (§ 3).

In our view, this approach to care ethics can be operationalized through an innovative account of two basic orientative principles and their systemic interrelation: autonomy, on the one hand, and the principle of vulnerability, on the other (§ 4). If successful, the practical implications of this approach pave the way for a revision of how care ethics is treated within the domain of engineering and design and subsequently a reimagination of how to translate these types of orientative principles into tangible design requirements. And this las point is a fundamental point with regards to the “design turn in applied ethics” (van den Hoven et al., 2017) given that the traditional top-down and bottom-up approaches have dominated the field of robotic design. Still, this paper does not delve into this issue, but rather provides the conceptual framework to springboard new discussions in engineering ethics for how to go about designing care robots according to the approach we discuss below.

## Issues and Approaches to Designing Care Robots

### The Top-Down Approach

This article aims to analyse how care robots–i.e., machines used in care practice–can be designed to support and promote the fundamental values in care practices. There is already a wide variety of autonomous machines currently used in assistance and care: My Spoon is a robot able to spoon-feed an assisted person, Sanyo, to wash and rinse him. Further, robots such as RIBA (Robot for Interactive Body Assistance) can move patients from one place to another, while Care-o-bots do likewise with objects in a room. And as well as robots to monitor people’s health and wellbeing, there are nursebots, used to reminds the elderly of certain routine activities (from eating and drinking to taking medicine and washing their teeth) and accompany their movements within a space–while Pepper, NAO, Kabochan, Brian 2.1, and Nexi 2 are humanoid robots that can not only move their arms, dance, and answer questions but also gather information through a camera and microphone and entertain the assisted person with basic games. This section intends to discuss the top-down and bottom-up strategies to design artificial moral agents (AMAs). “Top-down approaches to this task involve turning explicit theories of moral behaviour into algorithms. Bottom-up approaches involve attempts to train or evolve agents whose behaviour emulates morally praiseworthy human behaviour” (Allen et al., 2006: 149).

The “top-down” approach may look the easiest from an engineering perspective because it consists of programming the machine according to general behavioural principles (or laws). As noted by Van Rysewyk and Pontier (2015), such an approach is particularly apt to operationalize utilitarian or deontological ethical perspectives. It also follows a long-standing moral tradition that identifies the correct behaviour with that conforming to the law. Asimov’s three laws of robotics are an example of this type of solution, in that they bind the machine to act according to general principles at all times (Anderson, 2008). Some attempts to program robots to be “good” using a principlist approach have been reported in the literature. Winfield et al. (2014) discuss research in which robots are programmed to achieve a goal and to prevent other robots (as proxy humans) from getting hurt (for example, by falling into a hole or ending up in a dangerous area). According to Winfield et al. (2014, p. 5), this is an example of a robot that “appears to match remarkably well with Asimov’s first law of robotics: A robot may not injure a human being or, through inaction, allow a human being to come to harm.” Arkin (2009) have also have proposed a moral system able to adhere to the International law of war (LOW) and rules of engagement (ROE) and to distinguish between unethical and ethical actions based on their compliance or not with international law (Arkin 2009, p. 1).

The problem with a “top-down” approach is that the laws or general principles constitute overly generic moral references which may be hard to apply (or interpret) in complex real-life situations. Furthermore, each case is different and cannot be reduced *a priori* to law, which must be interpreted. Thus, an intelligent robot limiting itself to apply the instructions rigidly has received risks to interpret them inadequately and to the letter. For example, suppose we program a robot to serve,^1^ obey, and protect human beings. In that case, this could have disastrous consequences for humanity, in that they could think they are morally obliged to stop us from doing anything–because the less we do, the fewer chances we have of getting hurt–or to inflict serious cerebral damage on us as well, so that we perceive less pain.

Further, to safeguard and promote a patient’s wellbeing, a robot programmed to carry out care activities may feel justified in violating their personal sphere and refusing to obey them and meet their needs. For example, a robot could inform the health operators of their patient’s intention to put an end to their life, or not even help them die after an explicit request, even when the patient’s existence has become unbearable (Tonkens, 2015: 207–222). Furthermore, it merits questioning whether a care robot programmed to promote a patient’s good would stop the surgeon about to operate on them (Wallach and Allen, 2015: 92) or deceive them about their health when the prognosis is terminal.

Finally, the greater the number of rules and moral principles to be respected by the moral agent, the higher the risk that, in certain situations, two or more principles conflict. To this end, we should design autonomous machines to confront these situations and know what–in the case of conflict–is the principle that must prevail because it is “stronger.” That is, these types of dilemmas, or moral overload, create inextricable computational roadblocks. The way to resolve such issues computationally is to have higher-order principles that can be used to address such dilemmas (Allen et al., 2006: 150; Goodall, 2014). As new scenarios and new circumstances present themselves, we should add new moral rules and principles (that can resolve the existing conflicts) and specify their application. But this would not be a solution either. Even if it is always possible to determine when one norm has precedence over another, we cannot imagine all possible scenarios. “So even if deontological ethics can provide a guide in many situations, it cannot be used as a complete ethical system, due to the incompleteness of any group of rules and the difficulty of articulating human ethics in its complexity in a list of rules” (Goodall, 2014). That is, autonomous systems could end up in situations that were not foreseen nor even foreseeable. Moreover, even if we were able to formulate explicit criteria allowing an artificial moral agent (AMA) to override a rule, “any such criteria would very likely produce other dilemmas” (Wallach and Allen, 2015).

For this reason, it seems preferable to find/adopt a single moral principle or more general and abstract principles from which all other particular principles (or rules) can be derived. For example, utilitarian ethics stating that it is right to maximize happiness, wellbeing, preferences (or informed preferences), or pleasure for the greatest number of those involved. A strength of utilitarianism is its apparent ability to quantify goods and harms. The issue is that calculations could be highly complex and that an engineering model of ethics (i.e., do you have a dilemma? Apply the principle) is inadequate for moral life. Imagine, for example, that we wish to programme machines according to utilitarian ethics. We want “intelligent” care robots to promote the patients’ wellbeing as much as possible, but how can wellbeing be calculated? Only in terms of life years (the more, the better)? Or do we also consider economic, psychological, and social wellbeing? Significant disagreements could emerge regarding the objective to maximize, in that some would think it proper to maximize “happiness” while others would view it right to maximize “wellbeing.” Still, others may consider maximizing “preferences” (or “informed preferences”) while others again could give value just to “pleasure.”

Further, to calculate wellbeing, should we bear in mind only the most immediate consequences or take long-term effects (but how far away?) into consideration? This would also mean deciding which perspective the intelligent care robots should assume: whether to protect the interests of the owner or user (that is, the patient) at all times or consider a more stable, general point of view and protect the collective interest. Imagine, for example, that a patient finds themselves at home and is about to have guests over. Suddenly, a fire breaks out in their kitchen and spreads into the other rooms of the house: who must the care robot save? If we consider things morally, it seems fitting that the robot should not only worry about its owner but assume a general point of view: my wellbeing counts, but so does anyone else’s. However, still, certain decisions could end up as counterproductive. For example, programming care robots to protect general interests could reduce (or even extinguish) interest in these machines and slow down their adoption (at least in the short term). The most appropriate solution seems to minimize the consequences, considering the interests of both the care robot’s owners and anyone else (i.e., the general interest). But there is no single way to bring together and balance these different interests: we could give more value to the general interest, do the opposite, or consider them equally.

Utilitarianism presents the same problems as the so-called deontological ones. They appear to be unable to derive from highly abstract, generic premises; solutions can orientate people in concrete situations when choosing (Williams, 2011). Utilitarianism seems to permit the construction of scientific ethics. Still, the same precise and rigorous conditions cannot be achieved in practice as in science. Further, an engineering model of morality does not solve but intensifies moral contrasts in that it reduces all differences to divergences that are of principle disjunctions, hence harder to overcome. That is, “taking for granted that the only way to face ethical disputes is to apply this deductive and axiomatic model of practical rationality ends up making it almost impossible to overcome disagreement” (Lecaldano, 2005: 16).

### The Bottom-Up Approach

The alternative to the top-down model is the one we have called bottom-up: here, the strategy to make machines moral does not consist in giving them laws or general principles. The bottom-up approach allows artificial intelligence to learn morality (that is, what ethical behaviour is) through experience and learning without the need for general principles. Allen et al. (2006) liken this approach to the way a human child learns, and van Rysewyk and Pointier define it as an approach that creates “a series of learning situations through which a machine works its way toward a level of ethical understanding acceptable by the standards humans define” (2015: 99). Small pieces of knowledge gained through experience, manipulated by programmers as new challenges and tensions arise, all done within a learned social context in which the AMA is situated and able to grow (Allen et al., 2006: 151).

According to Aimee van Wynsberghe, the most promising manner of programming a care robot based on an alternative approach to the top-down one is to integrate the traditional value sensitive design (VSD) approach with normative criteria and elements from care ethics (van Wynsberghe, 2015; van Wynsberghe, 2016). Care ethics appears as an alternative perspective to the strategies inspiring a lot of modern moral philosophy based on an appeal to universal, abstract, and impersonal rules (principles that may be assumed to be valid for humanity overall), which should regulate the behaviour of separate, independent individuals. According to care ethics, it is wrong to reduce the moral to merely obeying norms and principles imposed on real life and people’s experience (Tronto, 1993; Botti, 2015; Collins, 2015). A long-standing philosophical tradition has linked morality with the ability to assume a completely detached perspective from our particular interests, the concreteness of the situation and relations, and the ties in which we are involved. Nevertheless, morality means not conforming to or applying general principles (or moral laws). Still, rather it corresponds to the ability to develop dispositions and practices of care and attention towards others (considered not as abstract individuals, but perceived in their concreteness and particularity). That is, from this perspective, morals are not the mere execution of a mechanical task (the simple application of a law or general principle to a particular case), but a practice requiring sensitivity (that is, attention)–and of course empathy–towards others. Likewise, it is also the subject’s awareness of their relation to others and developing an ability to listen and sentimental communication (Gilligan, 1982; Botti, 2015; Collins, 2015).

The considerations above should suffice to highlight that care ethics involves the importance of connection (Noddings, 1984; Noddings, 2002) and the relationship between individuals, their choices, and the context where they find themselves situated (Botti, 2018: 16). Dependence is the sign of vulnerability, but it is possible through this dependence to feel a responsibility of care towards one’s neighbour and to be able to pay more attention towards others (Gilligan, 1982; Noddings, 1984; Tronto, 1993; Held, 2006). Similarly, care is not perceived as a pre-set ethical perspective, ready for use in any context, but, as Wynsberghe (1,025) argues, it is a starting lens to recognize the other person’s dignity and begin to look after one’s neighbour. Care is already current practice in our lives, from birth to the moment of death (Noddings, 1984; Held, 2006). So, according to the supporters of ethical care, there is no need to justify it, but rather to take its importance into account to place it at the centre of morality (Botti, 2015).

In this view, starting from Gilligan’s original formulations, care ethics is not to be conceived as something which regards female subjects in as much as they can become mothers: it does not correspond to “maternal” ethics, but it does constitute a valid moral paradigm for any person (Slote, 2010; Slote, 2011). According to Virginia Held, care is first and foremost a form of emotional, reflective commitment (Held, 1993)–including sensitivity, solicitude, and worry, but also empathetic responsiveness, attention to specific needs and contexts, as well as relationships–which of course has a biological basis. However, this basis cannot be described as an animal- and/or human-type “instinct.” It is a social practice, a cultural transformation of something we already find in maternal care. Attention is sensitivity, which means that they are central aspects in responding to others’ needs. But these “natural” abilities may and (within an appropriate care process) must be refined and corrected through communication and dialogue exchanges (Held, 2006). Held does not dwell on this process of refining moral sensitivity. Still, from her perspective, emotions such as sympathy, empathy, sensitivity, and responsiveness are moral emotions that any individual should learn to cultivate to approach other people’s condition and do that which morality recommends. Maternal relationships may be the starting point for care activities, and, for Held, they remain an authoritative reference model. However, the activity of caring for another is only learnt through practice and experience. In this way, Slote (2010); Slote (2011) maintains, we may also develop a broadened, mediated empathy which stands in relation to situations not immediately present, through which we can even imagine the feelings of those farthest away.

In Joan C. Tronto’s opinion, care activity–“[o]n the most general level, we suggest that caring be viewed as a species activity that includes everything that we do to maintain, continue, and repair our “world” so that we can live in it as well as possible. That world, which includes our bodies, ourselves, and our environment, all of which we seek to interweave in a complex, life-sustaining web” (Tronto, 1993: 103)–plays out in four phases:

1. “Caring about,” where we recognize that care is necessary and we perceive the existence of a person’s need that must be satisfied. “Caring about–writes Tronto–will often involve assuming the position of another person or group to recognize their needs” (Tronto, 1993, p. 106).
2. “Taking care of,” that is, the moment in which we assume responsibility towards that need and consider what can be done, bearing the situation in mind. There is, then, recognition of the possibility of acting towards the identified need (Tronto, 1993: 106–107)
3. “Caregiving” is committing ourselves to satisfy the need through work requiring that the one giving care comes into contact with its recipients (Tronto, 1993: 107).
4. The fourth phase of the process or care activity is the “receiving care”, because care should also be measured in terms of appropriateness of the basis of the response from its recipient: “[u]nless we realize that the object cared for responds to the care received, we may ignore the existence of these dilemmas, and lose the ability to assess how adequately care is provided” (Tronto, 1993: 108).
5. There is, finally, a fifth phase of care–that is, *caring with*–which is specific to a democratic society in which the citizens are constantly involved in taking care, not individually–as autonomous, self-sufficient subjects–but together with other people as vulnerable subjects who need care and can trust and rely on other people. Care (the activity of care) is present in any society. Still, in a democratic society–writes Tronto–we have the best care activity because only in a democratic society is it possible to have institutions promoting caring *with* (Tronto, 2013; pp. 154–155).

These moments correspond to specific moral qualities: attentiveness, responsibility, competence, responsiveness, and trust (and solidarity). Attention is required because the caregiver must have the ability to perceive the continual changes in the situation and the needs of the person they are taking care of, in that there can be no care unless there is attention to others’ needs. Recognizing others’ needs is a challenging task, but this is precisely why it is a moral element, and ignoring others’ needs is without any doubt a moral evil (Tronto, 1993: 127), whilst responsibility is the ability to take on others’ perceived needs: it is not a promise; nor is it a commitment to act according to pre-set formal rules. In other terms, it is the ability to recognize that we must, based on the role we occupy and the skills we have, do something to change other peoples’ situation. Also, competence is the ability to consider the effectiveness of our actions, because “clearly, making certain that the caring work is done competently must be a moral aspect of care if the adequacy of the care given is to be a measure of the success of care” (Tronto, 1993:133). That is, Tronto states, truly responsible (and appreciable) cannot be uninterested in the consequences, because–she adds–“from a perspective of care, we would not permit individuals to escape from responsibility for their incompetence by claiming to adhere to a code of professional ethics” (Tronto, 1993: 134).

Then, responsiveness marks the importance of the care recipient’s response and the caregiver’s duty to pay attention to the “responses” of those cared for. Good care–writes Tronto–requires the four care process phases and appropriate integration of the different skills, or rather, moral elements necessary to perform it: “Such an integration of these parts of caring into a moral whole–states Tronto–is not simple. Care involves conflict; to resolve this conflict will require more than an injunction to be attentive, responsible, competent, and responsive” (Tronto, 1993: 136). Finally, trust results from people’s awareness that they can count on others’ participation in their care and care activities. At the same time, solidarity is built when citizens know that they can dispense care with others better (Tronto, 2013).

Following the care ethicist Joan Tronto (1993), Aimee van Wynsberghe identifies four fundamental values of care to be promoted in the design of (autonomous) systems: 1) attentiveness, as the capacity to recognize the needs of the care-receiver; 2) responsibility, which implies the caregiver’s concern for meeting the needs of the care-receiver; 3) competence, as the capacity of executing an action to fulfill the needs of the care-receiver; and 4) responsiveness or reciprocity, as the capacity of the care receiver to guide the caregiver and the instauration of a reciprocal interaction (van Wynsberghe, 2012; van Wynsberghe, 2013a; van Wynsberghe, 2013b; van Wynsberghe, 2016). Van Wynsberghe insists that these four elements are crucial in any care practice that impacts caregivers and care receivers due to the ethical importance they assign to the relationship and distribution of roles and responsibilities (Tronto, 2010). We may add a fifth element, including trust and solidarity as the caregiver’s ability to collaborate with others, in a democratic society, in care activity, enjoying trust in the willingness to participate, and collaborate with other people (van Wynsberghe, 2021).

### Artificial Morality and Moral Training

What we are asking is how to make care robots sufficiently moral, a question that has been at the centre of ethical concerns already for some time and widely disseminated by individuals like David Gunkel, who has reconstructed the different perspectives developed in philosophy over the relation between morality, artificial intelligence and robotics (Gunkel, 2012). Building on what we have discussed thus far, we can conclude that their design must consider both attentiveness and empathy in whichever care practice setting.

However, robots are machines: we can make them increasingly intelligent (and hence able to respond–or to react–to stimuli of human beings more and more appropriately), but they remain incapable–at least for the moment–of “sympathizing” with others’ needs and interests. So, this questions the possibility of building robots able to perform care activity integrating a traditional VSD (Value Sensitive Design) approach with elements from a care ethics perspective. Starting from Gilligan, care ethics has underlined that appropriate care activity does not consist of the ability to detach or abstract ourselves from the particular context or take distance from the actual features rationally, but instead of developing sensitivity and solicitude towards other people. And it is that attention to others’ specific, particular needs, of which Tronto also speaks, which is, in this view, reached through practice, experience, and specific abilities: the willingness to listen to the other and communicate with them (Tronto, 2013; Tronto, 2015). But, as Gilligan states (1982), sentimental communication, that is, empathy or sympathy, is also needed (Noddings, 1984; Noddings, 2002). Meaning the ability to let ourselves be influenced by other people’s emotions and feelings. For this reason, robots’ lack of any moral sensitivity seems to exclude from the start the possibility of attributing a minimal form of moral ability to them (Dumouchel and Damiano, 2017).

Yet, the fact that robots are not capable–at least at present–of feeling sympathy is not problematic: as suggested by Coeckelbergh (2020), while against the wishes of both cognitivists and feeling theorists of emotions until robots will have a consciousness, they will not be able to feel emotions properly, what Coeckelbergh calls “the appearance of emotions” and of being entirely moral can be attained. This is because we can program such sentiments synthetically rather than biologically. Even though others’ feelings cannot substantially influence a machine, it could learn to modify its behaviour and emotions based on others’ reactions and approval (or disapproval), developing this way a synthetic kind of nature-nurture interaction which resembles that building up to human moral development (Coeckelbergh, 2020). In this way, we may expect it to become a moral (or virtuous) machine over time; able, that is, to take into account not only the present situation and people but also the needs and interests of those it interacts and relates with, taken on in their particularity. The sentiments of love (or esteem) and hate (or contempt) for our fellows–David Hume (2007) and Adam Smith (1976) outline in their works–are the most potent motors of morality, in that we want others to like us and appreciate our behaviour and the passions and sentiments we have (Baier, 1991). Robots might be intelligent, but they are insensitive to others’ reactions because they are not conscious. However, they could be programmed to regulate their behaviour (and their “sentiments”) based on the esteem or contempt they receive from others. For example, moral approval (expressed by human beings) could be a reason to repeat specific behaviour, and moral disapproval a cause to modify/change it or make it more acceptable. We could call this ability to adapt “synthetic sensitivity” in that, like “biological” sensitivity, it denotes a disposition to put oneself in tune with the sentiments of other people, which is, as van Wynsberghe recalls, the essential quality for a good care provider: “Being in tune with the delicacy of the situation, and how to address it, can also be referred to as ethical sensitivity or “tinkering.” The former adheres to the idea of care as caring about while the latter is closely linked with care as caring for, albeit they are not mutually exclusive” (van Wynsberghe, 2015: 35).

Stating that robots can only become moral through practice and not thanks to abstract training simulations like those proposed in the Silicon Coppélia experiment mentioned by Van Rysewyk and Pontier, (2015)– find themselves, that is, interacting with people and becoming subject to their moral evaluation–means suggesting a different model of morality from that indicated by those who think it is possible to make a machine moral simply by programming it to obey certain principles.

This is not a defect but a strength in the approach we are suggesting. It recognizes the importance of experience and education for moral training and the inappropriateness of reducing the complexity of moral life to a few principles.

As announced at the very beginning, in our view, it is possible to integrate a 1) care-ethical perspective based on the consideration of particular care relationships, their contextual levels and the importance of needs, emotions, and sympathetic modes of deliberation with 2) a principlist approach to care. These two perspectives are not mutually exclusive, as it has been thought. They contribute to identifying a more comprehensive account of care practices that can be operationalized through an innovative interpretation of two fundamental and orientative principles and their systemic interrelation: the principle of vulnerability, on the one hand, and the principle of autonomy, on the other.

In Tronto’s words, care is not only an activity, but also a flair: “we insist that the activity of caring is largely defined culturally, and will vary among different cultures. Fourth, we see caring as ongoing. Care can characterize a single activity, or it can describe a process. In this regard, caring is not simply a cerebral concern or a character trait but the concern of living, active humans engaged in the processes of everyday living. Care is both a practice and a disposition” (Tronto, 1993: 103–104). For the Aristotelian ethics of virtue, dispositions appear as functions or abilities belonging to human nature.

In contrast, sentimentalist ethics consider the dispositions as individual character traits that are subject to approval or disapproval due to the consequences they produce. Referring, then, to Julia Driver’s definition, we can state that, “A character trait is a moral virtue if it is a disposition to produce (i.e., it tends to produce) intentional action that is systematically productive of the good (Driver, 2001:107). In other terms, dispositions are those personality or character traits that do not end in action because they represent principles, that is, stable conduct motifs (Baier, 1991; Baier, 1995): so we may call them qualities characterizing a person’s character or mind. (Hume, 2007: 3.2.1.2.).

A psychological disposition is made up of accepting a distinctive fan of considerations as reasons for action and a tendency to have a certain feeling or combination of emotions, often driving us to action. A robot cannot be moved by certain feelings–nor by combining feelings and passions–but it can still be programmed to act based on particular orientative principles and consequently manifest the disposition to behave in the way we prefer. On the basis, that is, of the ethical conception we are referring to, the ideal would be to have a care robot with the necessary sensitivity to respond appropriately to the feelings and emotions of the people he is called to care for. Indeed, for a robot to empathize with the people it interacts with, it would be easier to establish how to discharge its tasks. Yet, at least for the moment, hoping to build a robot endowed with sensitivity and our empathetic ability is unthinkable. Given the impossibility of counting on a compassionate robot, we can–as we have already said–consider making it synthetically (or artificially) empathetic through a programme allowing it to respond considering others’ judgement. But beyond this, programming it with orientative principles, we could also attribute to it a disposition “to be interested, look after and provide care when there is an unsatisfied need”. If the robot could have a character, we would not need to programme it with principles: but the robot cannot have a character, so the orientative principles could allow us to influence/condition its character sufficiently appropriately for our needs. (Dumouchel and Damiano, 2017). From a practical perspective, programming a machine to follow a few orientative principles could be advantageous. It would permit not only to control the machine’s behaviour but also to limit its autonomous space.

Furthermore, the robot would not need to learn to behave from scratch, in that it would already be programmed to follow certain principles, hence ways of behaviour (Allen et al., 2006; Wallach and Allen, 2015: 114–115). Nor would there be the problem characterizing “bottom-up” learning, which can be a prolonged, mistake-ridden process (Van Rysewyk and Pontier, 2015). There is still the risk that the overall principles are too general or poorly interpreted or that the robot does not know how to behave. Yet, in the terms described above, a machine sensitive to the reactions and responses of those it interacts with would be less subject to these problems. It could learn from practice and experience to correct its behavior. So even though it may misapply the moral principles, it could still always correct itself, taking into account the reactions of those it interacts with. Both Held (1993), Held (2006), Tronto (1993) stress the difficulty in grasping other people’s need for care, and for this reason, they emphasize the importance of dialogue and communication, as well as–naturally–the finetuning of our empathic abilities. Our capacity to take care of others’ interests and needs is limited: sometimes we do not perceive their suffering nor realize that we are causing them harm; certain forms or ways of life are invisible or at least remain opaque. A robot could ‘be born’ with our same defects, but, like us, could still, through experience/practice, become an appreciable “person”.This demonstrates that it is possible, in the case of robots, to integrate 1) a care-ethical perspective based on the consideration of particular care relationships, their contextual levels and the importance of needs, emotions and sympathetic modes of deliberation with 2) a principlist approach to care. These two perspectives are not mutually exclusive, as it has been thought, and contribute to individuate a more organic account of care practices which can be operationalized through an innovative understanding of two basic principles and their systemic interrelation: the principle of vulnerability, on the one hand, and the principle of autonomy, on the other.

## Fundamental Orientative Principles Within and Beyond the Care Ethics Approach

### Preliminary Orientative Lines

Based on the premises laid out in the preceding section, the main research question can be rephrased: how can we formulate a comprehensive approach that can frame the human-robot interaction overcoming the objective difficulties discussed above in terms of empathy? We are used to communicating such issues both from the point of view of human beings and machines. If we take the first issue discussed in the preceding section, what we are doing is referring to the possibility–in some cases welcomed, in others indeed feared–that specific groups of human beings might develop feelings for robots. More specifically, there is a typology of relationships that emerge, i.e., by persons with mental impairments or by elderly people with affective difficulties or, still, by persons addicted to robot companion and/or sex robots (Sharkey, 2014; Bendel, 2017; Balistreri, 2018; Ostrowski et al., 2019; Bisconti Lucidi and Piermattei, 2020; Jecker, 2020).

On the other hand, we refer to the objective difficulty of human-robot interaction by considering the second viewpoint: the tension between a robot executing a programmed behaviour and an empathetic behaviour towards a human subject. Of course, we can consider a situation in which the programmer is committed to programming a sort of *synthetic empathy* that can resemble or replicate, as much as possible, empathic behaviour that is observed in human-human interactions. Again, we are aware that this is one of the most promising frontiers of the intersection of big data enquiry and artificial intelligence (Cavallo et al., 2018).

We are thus perfectly aware of the existing and expanding debate on such issues. Nonetheless, it is not necessarily to expand on what are indeed promising theoretical directions in what follows. Instead, we would aim to propose a preliminary outline of what has been explored up to now. This is a sort of theoretical framework that, in a sense, acts as a foundation, and that thus we propose as one to be inserted into the care-ethics approach we presented above.

The final expected goal of this insertion (and of the entire research project which this paper is part of) is to propose a renewed care-ethics approach that shall integrate the VSD approach articulated by van Wynsberghe. In what follows, we will offer a comprehensive argument to ground the legitimacy of such a theoretical framework by justifying the insertion of two basic principles whose argumentative role is identifying an avenue along which trying to integrate a possible renewed program in care ethics–we attempted to articulate a preliminary attempt to frame the implementation issue of an integrated VSD in Umbrello et al. (2021). In turn, we would like to deepen the same linkage between that framework and the more traditional care ethics approach in a further step.

### Methodological Remarks

In approaching such a framework, some preliminary and methodological remarks should be clarified.

Firstly, in line with the objections expressed above (§ 2.1), it merits reiterating that referring to two principles does not mean that we implicitly affirm a top-down approach. We remain convinced that such a top-down approach risks substantiating nothing other than general principles that do not offer concrete guidance in specific situations or actual settings.

Secondly, introducing a unique principle might imply operationalizing such a principle by underestimating the contradictory or aporetic effects derived from it. Some of them, related to the utilitarian principle of happiness, have been analyzed above. This tension can be exacerbated by introducing other relevant principles like “dignity” or “respect”, for example. One can already imagine the conflict that would undoubtedly emerge through a haphazard combination of two or more principles. A striking exemplar would be between managing the moral overload between two principles like happiness and dignity.

Furthermore, introducing several (all fundamental) principles can’t exclude the possibility of creating conflicts and dilemmas that are undoubtedly difficult to solve within specific operational situations. This is already true for human beings, but it remains even more evident for robots. Thus, being fully aware of the risks of implementation and the conflicts related to a top-down approach, we indicate two principles that must be framed not as antithetical but rather complementary.

To methodologically avoid affirming a top-down approach, we instead actively aim at constructing a *revision* of a bottom-up approach. This, of course, does not mutually exclude other approaches like the so-called “mid-level ethical theories” that have been proposed by van den Hoven (2010), Jacobs and Huldtgren (2018), and Cenci and Cawthorne (2020) following the theoretical path traced by the likes of Martha Nussbaum, Amartya Sen, and John Rawls. We have elsewhere employed such an approach in application to care robots (Umbrello et al., 2021). What characterizes this revision of a bottom-up approach is marked by a straightforward adoption of theories of relationality as they pertain directly to vulnerability and autonomy, something that those other approaches do not undertake. That is the line of argumentation that will be followed here. Nonetheless, some methodological remarks need to be explained in a revised bottom-up approach as the one we aim to construct here.

We introduce four remarks here. The first remark is related to the specific theoretical usage of the term “principle.” Selecting care ethics as the primary focus implies some particular difficulties in inserting a discourse on principles since care ethics originated from the need to avoid an “ethical principle” in the traditional sense of the term. However, the meaning of the word “principle” we suggest and deem appropriate here is not to be understood as a normative term that is external/independent to the situation and subsequently commands in the universalistic sense of the word. Simply put, we are not thinking of a principle that reproduces the same normative constraints that the Kantian categorical imperative entails.

Instead, and like Kant, we envision the usage of the term “principle” as analogous to the one he employed in the *Critique of Pure Reason*, where he refers to the “transcendental ideas” that pure reason faces in the “Transcendental Dialectic” (Kant, 1998, 394–408). Here he argues that these are not to be understood as strictly “binding principles”; rather, they are instead “orientative principles.”

That expression refers to principles that must be considered asymptotic lines that can aggregate and gather patterns of behaviours that might *prima facie* be considered divergent. They are the peak of normative purity that trigger a constant interest for human reason and which individual agency is inescapably addressed. In Kantian terms, they are principles that cannot be the object of knowledge. Principles, then, are impossible for human beings to experience. We do not know such principles nor experience them in the total sense of the term. We can rationally imagine them as a point of orientation, a focus that orients our action and systematizes it along a coherent path.^2^

If we defer to the Kantian perspective, this comprehensive qualification of the “transcendental ideas” as “principles” is valid for both the cognitive and the practical realm. In the present context, we can imagine the same extended perspective. Nonetheless, what is more relevant for this level of discussion–and this is the second methodological remark–is that those principles should be considered orientative for and within any context of interrelation. The implications of this point are twofold.

1. On the one hand, we affirm that those principles should be considered orientative lines for any possible setting or state of affairs in which a form of exchange occurs that we can call “interaction.” Phenomenologically speaking, we might agree on a minimal meaning of the term “interaction” by affirming the coexistence of two conditions: 1) this term describes a univocal spacetime frame in which at least two agents are present; 2) they are or become aware of the (effects of an) action that the first is doing as addressed to the second.
2. On the other hand, we would like to suggest that those principles should orient the pattern of action that guides any possible relationship among beings that share a “status of subjectivity.” As a primary point of reference that can be commonly shared, with this expression, we mean a being that can start a “state of affairs” they are responsible for. We don’t want to enter here further by embarking on a theoretical account of the subjectivity of robots [for a preliminary framing, see Stradella et al. (2012)]. Taking for granted that we are speaking about robots that are complex enough to consider themselves as starting points of possible (patterns of) actions, we are just alluding to the possible relationship network of humans-humans and humans-machines (by leaving open the possibility to imagine robot-robot relationships–which we do not investigate here).

Thirdly, by considering the principles we will articulate as orientative ones, we are paving the way for a different line of inquiry. We are thinking of principles that are intense, pervasive, and flexible enough to both inform and orient at the same time, as well as function in any context of the interrelation of each possible “subject” involved in it–again, by attributing such a status to both humans and robots. In more synthetic terms, we maintain that such a kind of principle can be adequate for orienting both the contextual settings or situation (embedding perspective) and the forms of subjectivity involved in them (embodying perspective). We will integrate this point in the following paragraph.

Last but not least, as our fourth methodological remark, the comprehensive approach here used can be described as hermeneutical. We can attribute this evocative word to the same twofold meaning that Hans-Georg Gadamer intended (Gadamer, 2013)–“fusions of horizons” on the one hand and as “history of effects” on the other. We would like to allude that any single situation or context of interrelation has its characteristics, spatial/temporal borders and constraints, and it embeds specific actors in it. There are no privileged or external points of view that can account for it with sufficient accuracy–and, consequently–that can take appropriate decisions outside the subjects involved in it. If this affirmation may be considered accurate for any interaction situation, it becomes still more evident by referring to forms of relationships mediated by technologies (Pirni and Carnevale, 2013; Pirni and Carnevale , 2014; Pirni et al., 2017). Again, this is another way of excluding from the very beginning the possibility for any top-down (now: external-internal) approach. Moreover, accuracy in making decisions is in direct proportion to the habits of interrelating with those specific agents or issues. In short, it is the result of intersubjective historicity that is lived in common.

## Autonomy and Vulnerability: A Duality of Principles for a Renewed Care-Ethics Approach

By moving forward in designing the proposed approach, we must outline a comprehensive definition of each of the two principles we want to offer in this context. Again, to avert any reference to a top-down approach, we avoid the insertion of any unique or unifying principle. Instead, the insertion of the systemic linkage of two orientative principles (in the sense outlined above) we are going to illustrate has to be considered within a sharp argumentative lie that can be summarized in what follows:

1. Both principles are to be considered on the same plane or level. No one of them should be regarded as a priority, even in extreme conflictual situations;
2. They are constitutively complementary: neither an account of (a focus on) autonomy without considering vulnerability nor an asymmetric opposite account (focus) is admitted. They are two, but none of them can be neither subjected to the other nor avoided or underestimated in favour of the other.

### Autonomy and Care: A Preliminary Outline

Accordingly, the comprehensive account of each of the two principles should be considered within a strong linkage with the other. This is precisely the meaning through which the same hermeneutic/phenomenological framework outlined above articulates. If we try and grasp the most phenomenologically evident meaning of both principles, we can affirm that they are opposed. What we are alluding to here is a theoretical understanding, which is related to an experience from the first-person point of view, more than linguistic antonyms (the subsequent paragraphs are devoted to expand and widely ground this point). Rather than being a weakness of our argument, their constituting opposite principles are, in fact, an intended characteristic that substantiates our reasoning. By adopting both principles, on the one hand, through *autonomy*, we set as an objective the guarantee of the maximum conceivable extent of independence to the individual. At the same time, by also contemplating the principle of *vulnerability*, we aim at the full possible degree of relationality and dependence of a subject from (an)other one (-s).

Accordingly, to try and outline a meaning of the principle of *autonomy* that is constitutively open towards and provide a potential integration to the principle of *vulnerability*, certainly the common understanding of “autonomy” must be finetuned in some of its primary and “classic” characteristics.

First of all, we must distinguish *autonomy* from *arbitrium*, distinguishing the former from arbitrary/unconstrained agency. Being autonomous does not mean “to do whatever one wants” nor whatever is conceivable and possible, according to one’s own overall capability to act/to avoid acting in any context in which one’s action might take place and be oriented to any (subject or thing) present in it. Such a definition would correspond to the very meaning of the concept of *arbitrium*. Here we indeed wish to differentiate from the first principle we were introducing-that is autonomy.

Instead, the concept of autonomy we are searching for is a principle that systematically opens up the possibility of acting in a context in which other subjects are acting or might act and, therefore, endowed with a *relational* dimension. In this understanding, the individual will is structurally open to any possible principle of concrete acting. Yet, it must select and choose among possible principles of acting while keeping in mind a general meta-rule that is shaped in line with the Kantian third formulation of the categorical imperative. This imperative would mean that anytime you are on the verge of acting, try to articulate, select, and put into practice just. Only that principle of action can you rationally imagine that any other subject may want and affirm on their behalf. In other words, try to act by *orienting* your agency while having in mind a systemic approval of your action and the subjective principle that guided it by any other subject who might act or might be the recipient of your activity in the same context. As we can see, such a formulation of the categorical imperative is inextricably tied to a conception of the individual, which is far from being solipsistic, like other different understandings of the concept of autonomy suggest.

Of course, in line with a relational conception of subjectivity, the target of our conceptual framework corresponds both to the individual as the subject who acts and to the individual as the subject who “receives” the action. In this perspective, then, being autonomous does not mean being a solipsistic agent pursuing their own goal whatever the conditions, whatever other subjects and correlative goals they may encounter. Rather, being autonomous means finding a systemic and dynamic balance between the need for self-sufficiency and the capability to start a state of affairs by one’s own will on the one hand, and the need to take care of the analogous need and capability that guides and orientates the agency of any other subject in any specific context, on the other.

Ultimately, according to this definition, being autonomous means taking care of the autonomy of others as well as of the potential fragility and vulnerability that is endowed in each and every one of us (Pirni, 2006; Pirni, 2013; Pirni, 2016).

### Vulnerability and Care: A Preliminary Outline

Given the understanding of autonomy we just outlined, its interplay with the principle of vulnerability is less problematic than one would expect. The endorsement of vulnerability can now be understood as coherent with but even necessary to the realization of the idea of autonomy we set up in the previous section. To define vulnerability, as a first approximation, we can build on a phenomenologically evident (instead of at-first-sight contradictory, as with our definition of autonomy) understanding of the concept. According to this understanding of vulnerability, the basic situation becomes one in which no individual can either live or survive, nor can they pursue their own goals alone. Relationality, in this view, is not just courtesy, nor a possible or socially acceptable behaviour, but rather an intrinsic characteristic of the subject. Such subjects do not stand alone but are permanently embedded in a relational net with others for survival and fulfilment. Relationality is a matter of systemic and vital necessity.

It merits noting here that we are not alluding to unique situations that bear clear evidence, like the condition of people with impairments. Instead, the perspective we adopt is in line with theories that see subjects as embedded in a net of relations and in contextual circumstances that try to manage - but, in the end, are forced to confirm-our constitutive dependence at the individual level as well as at the systemic one (Kittay, 1999). Such perspective is shared by normative frameworks such as relational egalitarianism (See Voigt, 2020). More importantly, the present research is first and foremost built upon the understanding of subjectivity expounded by feminist thinking (See, *inter alia*, Butler, 2004). Thus, we are not referring to a vulnerable subject in the sense that they are practically dependent on others to fulfill daily needs or perform basic activities. Instead, we refer to the basic constitutive situation of vulnerability shared by every human being as an embedded feature of humanity, which is accurate and operating for each subject capable of acting. Such a subject is solely *prima facie* independent or autonomous. They are forced to act in a world shared with other subjects, within definite boundaries and facing a series of limitations in terms of lack of resources or deficiency of time. Following Arendt (1958), we can say that “Men [human beings] and not Man, live on the earth and inhabit the world” and that one defining characteristic of humanity is plurality. Further, they can be cognitively/ethically vulnerable, in the sense of not being equipped with sufficient knowledge as well as the ethical competencies to overcome specific difficulties, constraints, and limitations that interfere with both the most linear pursuit of their tasks and the due care to the autonomy/vulnerability of others.

In sum, the final achievement of this provisional theoretical path is a constitutive interdependence between the two principles. This might offer a challenging and potentially open theoretical “platform” to relaunch a care ethics perspective more in line with the demanding and urgent reshaping of any possible integration between human and machine.

## Conclusion

Various approaches have been undertaken in an attempt to integrate ethical principles and practices in care ethics. This has similarly been an approach applied to the design and development of robotic technologies that fall within the domain of care (§ 1). This paper has taken these approaches as a starting point, illustrating how they have been employed and their shortcomings. In particular, we showed how both the traditional top-down and bottom-up approaches have fundamental misgivings (§ 2). This, consequently, is inextricably linked with foundational ethical issues. To address these issues, we propose a revision of the bottom-up approach as the most salient starting point for rethinking care ethics as it is applied to robots. The central innovative contribution of this paper is the proposal of rehabilitation of two orientative principles that can surround the entire theoretical building of any care ethics approach.

These principles were selected following a specific methodology (§ 3), which led to identifying an ethical horizon where the interplay between autonomy and vulnerability includes both humans and machines on a single plane. On the one hand, this horizon enhances the potential autonomy of both, but it also highlights their respective and constitutive vulnerability. On the other, this opens up the possibility of a new relational dimension (§ 4). In doing so, the central contribution of this approach aims to provide a framework that promises a more salient interplay, and possibly a novel integration, that is directed towards the future of our “living togetherness,”

## Data Availability

The raw data supporting the conclusions of this article will be made available by the authors, without undue reservation.

## Footnotes

1 Which can also be understood in the negative sense, i.e., allowing for a certain degree of (negative) freedom to do risky things.

2 For a parallel approach that uses “insight” as the key-concept instead of “principle,” in order to give space to a similar need for an orientative perspective in care ethics, see Leget et al. (2019).
